# Miscellany

**Published:** 1896-06

**Authors:** 


### Miscellany.

The Wilhelm Meyer Memorial.—In recognition of his great services to humanity it is proposed to erect a monument to the late Prof. Wilhelm Meyer, in his native city, Copenhagen, Denmark. To this end committees have been formed in all the principal countries of Europe for the purpose of raising the necessary funds. A large and influential national committee, representative of all the leading sections of this country, has been appointed and power given to each member of it to make such local arrangements as in his judgment shall best secure the success of his work. It is hoped that all who have profited by the results of Dr. Meyer's teachings, not only of the medical profession, especially in the departments of laryngology, otology and pediatrics, but also among the laity, will be willing to aid in the accomplishment of the object for which the committee was formed. F. W. Hinkel, M. D., is the member of the committee for Buffalo, and to him any contributions may be made.

---

The William F. Jenks Memorial Prize.—The fourth triennial prize of $400, under the deed of trust of Mrs. William F. Jenks, will be awarded to the author of the best essay on The etiology and pathology of diseases of the endometrium, including the septic inflammations of the puerperium. The essay, which must be written in the English language, or if in a foreign language accompanied by an English translation, must be sent to the College of Physicians of Philadelphia before January 1, 1898, addressed to Barton Cooke Hirst, M. D. Each essay must be typewritten, distinguished by a motto and accompanied by a sealed envelope bearing the same motto and containing the name and address of the writer.

